# Erythrocytic α-Synuclein in Parkinson’s Disease and Progressive Supranuclear Palsy—A Pilot Study

**DOI:** 10.3390/biomedicines12112510

**Published:** 2024-11-02

**Authors:** Costanza Maria Cristiani, Luana Scaramuzzino, Elvira Immacolata Parrotta, Giovanni Cuda, Aldo Quattrone, Andrea Quattrone

**Affiliations:** 1 Neuroscience Research Center, Department of Medical and Surgical Sciences, University “Magna Graecia”, 88100 Catanzaro, Italy; 2 Institute of Molecular Biology, Department of Medical and Surgical Sciences, University “Magna Graecia”, 88100 Catanzaro, Italy; 3 Research Centre for Advanced Biochemistry and Molecular Biology, Department of Clinical and Experimental Medicine, University “Magna Graecia”, 88100 Catanzaro, Italy; 4 Institute of Neurology, Department of Medical and Surgical Sciences, University “Magna Graecia”, 88100 Catanzaro, Italy

**Keywords:** Parkinson’s disease, progressive supranuclear palsy, α-synuclein, oligomeric α-synuclein, phosphorylated α-synuclein, red blood cells

## Abstract

**Background/Objectives:** The current research examines the accuracy of α-synuclein in RBCs as a diagnostic biomarker for PD and PSP, despite their distinct molecular etiologies. **Methods:** We used ELISA to measure total, oligomeric, and p129-α-synuclein levels in erythrocytes from 8 PSP patients, 19 PD patients, and 18 healthy controls (HCs). The classification performances of RBC α-synuclein levels were investigated by receiver operator characteristic (ROC) curve. We also evaluated a possible correlation between RBC α-synuclein level and the biological and clinical features of our cohorts. **Results:** RBC total α-synuclein was higher in PSP patients compared to both PD patients and HCs, achieving good classification performance (AUC: 0.853) in distinguishing PSP patients from PD patients, with a sensitivity of 100% and a specificity of 70.6%; moreover, the levels of this biomarker positively correlated with disease severity in PSP group. Regarding oligomeric α-synuclein and p129-α-synuclein, the latter was slightly increased in RBCs from PSP patients compared to HCs, but no correlations were detected. **Conclusions:** Although these findings need to be confirmed in larger studies, our pilot work suggests that RBC total α-synuclein may represent a potential molecular biomarker for the differential diagnosis and clinical staging of PSP.

## 1. Introduction

Parkinson’s Disease (PD) is the most common form of parkinsonism, a group of neurodegenerative diseases characterized by rest tremor, bradykinesia, rigidity, and postural and gait impairment [1]. At the molecular level, PD is a synucleinopathy whose major pathological hallmark is the presence of Lewy bodies, which are insoluble protein inclusions mainly composed of aggregated α-synuclein [2]. The α-synuclein is a small 140 aa protein mainly located at presynaptic terminals that can assume multiple physiological conformations, ranging from the cytosolic unfolded monomer to the α-helical membrane-bound form [3]. The structure of α-synuclein can also switch to a β-sheet conformation in response to environmental cues such as changes in pH, ions, and metal concentration. In this form, α-synuclein firstly self-aggregates in soluble oligomers (o-α-synuclein), considered as the cytotoxic form of the protein, and then into insoluble fibrils forming the histopathological inclusions [2,4]. The process is further induced and enhanced by post-translational modifications occurring in α-synuclein protein. Among them, phosphorylation at Ser129 (p129-α-synuclein) is the most known to induce aggregation [5]; indeed, p129-α-synuclein accounts for 90% of the insoluble protein found in PD lesions [6].

The role played by α-synuclein in PD pathogenesis has prompted the investigation of this protein in biological fluids as a biomarker. α-synuclein measured in CSF was consistently lower and o-α-synuclein and p129-α-synuclein were consistently higher in PD patients compared to healthy controls (HCs) [7,8,9], while evidence of its presence in serum and plasma has been more conflicting so far [10,11,12,13,14,15,16,17].

However, the most abundant blood source of α-synuclein is red blood cells (RBCs) [18], suggesting they may represent a valuable and more reliable source for this biomarker. Indeed, it has been suggested that discrepancies in peripheral α-synuclein levels observed among studies may be attributable to α-synuclein release from RBCs during hemolysis [10]. Moreover, erythrocytes have been shown to be altered in many neurodegenerative disorders, displaying changes in their morphology, metabolism, and protein expression as well as increased oxidative stress [19]. Particularly, previous studies have showed that PD patients had higher levels of total, oligomeric, and p129-phosphorylated protein in RBCs compared to control subjects [20,21,22,23,24,25,26]. In addition, RBC α-synuclein has been demonstrated to help in distinguishing PD from essential tremor [23], which suggests that α-synuclein could also be useful in discriminating between movement disorders.

Progressive supranuclear palsy (PSP) is an atypical parkinsonism showing a remarkable overlap in symptomatology with PD, especially at the early stage of the disease [27], representing an important challenge for clinical diagnosis. α-synuclein may be a useful biomarker to discriminate between PD and PSP, due to the key differences in the molecular bases of these two diseases. However, α-synuclein assessment in biological fluids showed inconclusive results in previous studies [14,28,29,30,31,32]. Nevertheless, a precise diagnosis would be highly valuable to improve patient management as well as stratification for clinical trials.

In the present work, we investigated the usefulness of RBC total, oligomeric, and Ser129-phosphorylated α-synuclein in discriminating between PSP and PD, aiming to identify a blood diagnostic marker based on ELISA technology, which is widely available in clinical practice. The individuation of an easily accessible and assessable biomarker distinguishing between PD and PSP could be rapidly applied in clinical routine and would be highly valuable for a precise diagnosis, which in turn would improve patient management as well as stratification for clinical trials.

## 2. Materials and Methods

### 2.1. Subjects

Eight PSP and nineteen PD patients fulfilling the MDS diagnostic criteria [1,27] were recruited at the Movement Disorder Center of the Magna Graecia University of Catanzaro. Of the 8 PSP patients, 1 was diagnosed with probable PSP-Parkinsonism (PSP-P), 1 was diagnosed with probable PSP with progressive gait freezing (PSP-PGF), 2 were diagnosed with possible PSP with predominant corticobasal syndrome (PSP-CBS), and 4 were diagnosed with probable PSP-Richardson’s Syndrome (PSP-RS). Normal striatal uptake on 123I-FP-CIT- SPECT and MRI signs suggestive of normal pressure hydrocephalus and clinical features suggestive of other diseases were considered as exclusion criteria for both patient groups. Neurological examinations, including MDS (Unified Parkinson’s Disease Rating Scale part III (MDS-UPDRS-III)) and the Hoehn and Yahr (HY) Staging Scale in off-state, were performed in all patients, and the PSP rating scale was used to further evaluate PSP patients [33]. As HCs, 18 subjects without any neurological or psychiatric disorders and without close relatives with neurodegenerative diseases were recruited. The study was performed according to the Declaration of Helsinki and approved by the Calabria Region Ethics Committee. All the involved subjects gave written informed consent for their participation in the study and the use of their medical records for research purposes.

### 2.2. RBC Collection and Biomarker Assessment

Whole blood from each subject was collected in BD Vacutainer^TM^ EDTA tubes collection (BD Biosciences, San Jose, CA, USA) between 9 a.m. and 12 p.m. and processed within 30 min from collection. Samples were centrifuged at 3000 rpm for 10 min at 4 °C to obtain stratification, then the upper and middle layers, composed of plasma, white blood cells, and platelets, were carefully removed. RBC pellet was then washed three times in phosphate-buffered saline (PBS) 1X, aliquoted, and stored at −80 °C until use.

For biomarkers assessment, frozen (−80 °C) RBC pellet aliquots were thawed at room temperature (18–23 °C) for 30 min to induce heat-shock-mediated cell lysis and centrifuged at 20,000 rcf for 30 min at 4 °C to remove cell debris. Hemoglobin was measured by spectrophotometry.

Total, oligomeric, and Ser129-phosphorylated α-synuclein were all evaluated by specific ELISA assays (MBS2602614, MBS043824, and MBS038716, respectively; MyBioSource, San Diego, CA, USA) on a Varioskan™ LUX multimode microplate reader (Thermo Fisher Scientific, Waltham, MA, USA). Manufacturer’s protocols were adapted by diluting RBC samples 1:100 in PBS 1X for total α-synuclein detection and 1:50 in PBS for oligomeric and Ser129-phosphorylated α-synuclein detection. All the measurements were performed in duplicates. Values were normalized by dividing the absolute α-synucleins concentration values for hemoglobin (Hb) level in the biological sample to minimize differences due to RBC numbers across subjects [34].

### 2.3. Statistical Analysis

All statistical analyses were performed by using IBM SPSS v29.0.1.0 (Armonk, NY, USA). Fisher’s exact test was used to assess differences in sex distribution. Normal distribution of continuous variables was tested by Shapiro–Wilk test. ANOVA was used to assess the differences in age between groups, Student’s *t*-test was used on disease duration and MDS-UPDRS-III, and the Mann–Whitney test was used for the HY Staging Scale. Differences in total, oligomeric, and p129-α-synuclein were assessed by ANCOVA with sex and age as covariates, followed by Tukey’s LSD post hoc test. The diagnostic value of α-synuclein was assessed by receiver operator characteristic (ROC) curve and Youden Index was used to select the cut-off, giving the maximum sum of sensitivity and specificity. A *p*-value < 0.05 was considered as significant for all the analyses.

## 3. Results

### 3.1. Demographic and Clinical Features

A total of 8 PSP patients, 19 PD patients, and 18 HCs were included in the study. The demographic and clinical features of the groups are summarized in Table 1. Compared to both the groups of patients, HCs showed a higher proportion of female subjects, while age range was comparable. Clinically, disease duration was comparable between PD and PSP patients, but the latter showed higher MDS-UPDRS-III and HY Staging Scale scores, in accordance with the more severe clinical phenotype of PSP [26].

### 3.2. α-Synuclein in RBCs

**Figure 1:**
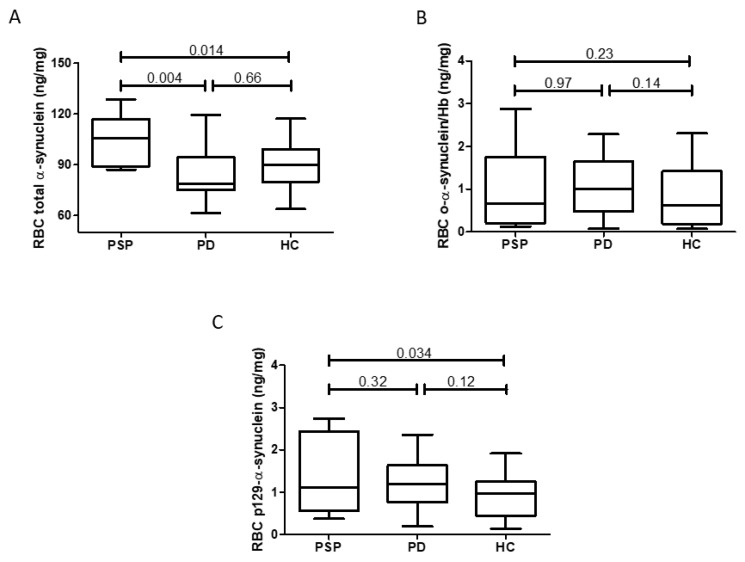
Erythrocytic concentration, corrected for Hb content, of total (**A**) oligomeric (**B**) and p129- α-synuclein (**C**) in PSP patients (*n* = 8), PD patients (*n* = 19), and HC (*n* = 18). Data are summarized as box plots, in which the lower, upper, and middle lines of boxes represent the 25th percentile, 75th percentile, and median, respectively, while limits of vertical lines indicate ranges. Shown *p*-values were obtained by ANCOVA with age and sex as covariates followed by Turkey’s LSD post hoc test. o-α-synuclein = oligomeric α-synuclein; PSP = progressive supranuclear palsy; PD = Parkinson’s disease; HC = healthy control.

Expression of total α-synuclein, o-α-synuclein, and p129-α-synuclein was detected in RBCs from all the three groups. Since our cohort showed a significant difference (*p*-value < 0.05) in sex distribution (Table 1), the analysis was performed correcting for age and sex. Among the three biomarkers, the total α-synuclein concentration showed a significant difference (*p* = 0.011), with a higher content in RBCs in PSP patients compared to both PD and HC. p129-α-synuclein also showed a significant difference between PSP patients and HCs but to a lesser degree than total α-synuclein (Figure 1 and Table 2). No differences were detected in Hb content between groups (Figure S1).

### 3.3. ROC Curves and Correlation Analysis

**Figure 2:**
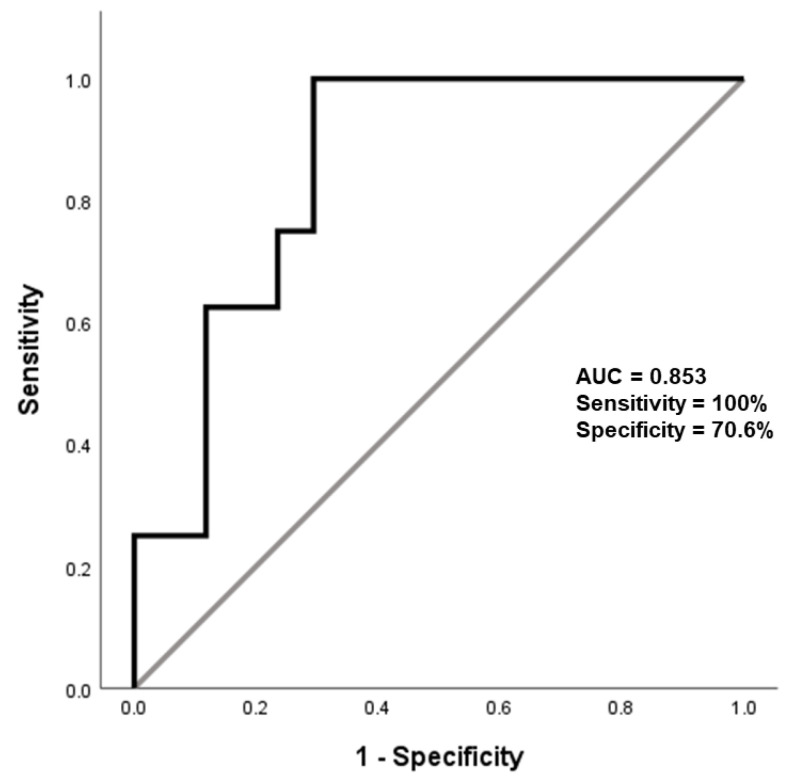
Performance of erythrocytic total α-synuclein/Hb in differentiating PSP from PD patients.

To evaluate the accuracy of total α-synuclein/Hb ratio as biomarker to discriminate PSP patients from PD patients, an ROC curve was constructed and the area under the curve (AUC) was calculated. The analysis showed that total α-synuclein/Hb ratio effectively distinguished PSP patients from PD patients (*p*-value: 0.004), with an AUC of 0.853, with a sensitivity of 100% (95% CI: 63.06–100%), a specificity of 70.6% (95% CI: 41.34–88.98%), and a value of 85.06 ng/mg total α-synuclein/Hb was set as the cut-off (Figure 2).

To further evaluate the possible association between α-synuclein and demographic and clinical features in our cohorts, Spearman’s correlation tests were performed. PSP Rating Scale and MDS-UPDRS-III were used for PSP and PD patients, respectively, to assess the associations with disease severity. Notably, total α-synuclein displayed a strong positive correlation with disease severity in PSP patients (Table 3). On the other hand, neither erythrocytic oligomeric nor p129-α-synuclein correlated with demographic or clinical parameters in the PD and HC groups (Table 3).

## 4. Discussion

RBCs are the most abundant cell component of blood, representing 40 to 45% of blood volume [35]. About 98% of RBC protein content is composed of hemoglobin, and the remaining 2% is largely uncharacterized [34]; however, among the non-hemoglobin proteins, RBCs largely express α-synuclein, exceeding 1000 times the concentration observed in plasma [18]. In the erythrocytes, α-synuclein is up-regulated during the late stages of differentiation, because of the role for this protein in enucleation and membrane stabilization [36,37,38], and RBCs retain α-synuclein upon maturity. In addition, erythrocytes have been suggested to uptake extracellular proteins [39,40]; therefore, the α-synuclein found in RBCs could also partially derive from external sources. Particularly, it has been demonstrated that erythrocytes can internalize α-synuclein oligomers through clathrin-dependent endocytosis, although the specific receptor involved has not been identified yet [41]. The two mechanisms are not mutually exclusive; therefore, α-synuclein in RBCs can have both intra- and extracellular origin.

In this work, we assessed for the first time the presence of total, oligomeric, and p129-α-synuclein in RBCs from PSP patients. We observed increased levels of total α-synuclein in erythrocytes of PSP patients compared to both PD and HC. A slight significant increase in p129-α-synuclein was also observed in the PSP patients in comparison with the HCs. Total α-synuclein effectively discriminated between PSP patients and PD patients (AUC: 0.853), with an excellent sensitivity (100%) and an overall good specificity (70.6%). Although this finding may sound counterintuitive, a previous study reported increased amounts of α-synuclein in plasma of PSP patients [42], supporting the peripheral accumulation of this protein in PSP.

The biological mechanism behind the increased presence of α-synuclein in PSP RBCs is unknown. One possible explanation could be related to the detrimental effects exerted by tau protein on blood–brain barrier function, which appear to be more aggressive compared to α-synuclein [43], causing a more pronounced protein leakage. In this context, RBCs might act as scavengers for α-synuclein, reducing the circulating levels.

The significant correlation we detected between erythrocytic total α-synuclein and disease severity corroborates this hypothesis. Thus, the α-synuclein increase detected in RBCs of PSP patients may suggest that a major part of the accumulated protein might be a physiological form deriving from dying neurons. Anyway, this mechanism is speculative and warrants further investigation.

Currently, the best peripheral biomarker in distinguishing between PSP and PD is the light chain of neurofilament (Nf-L), a marker of axonal damage [44,45] which has been shown to be very powerful as biomarker when combined with MRI parameters [46,47]. However, Nf-L has been found to be increased also in other neurodegenerative diseases [43,44], providing limited specificity. In this context, RBC total α-synuclein could represent a second, easily assessable biomarker that could be combined with Nf-L to increase the clinical performance of this protein.

In our study, we did not confirm previous evidence indicating an accumulation of α-synuclein in RBCs from PD patients compared to control subjects [20,21,22,23,24,25,26]. Notably, the previous findings were all obtained in Chinese cohorts, while we enrolled only Italian patients with Caucasian origin. PD is a multifactorial disease caused by the interaction between genetic variants and environmental factors, such as HCV and *H. pylori* infections, changes in intestinal microbiota composition, and exposure to metals, pesticides, and pollutants, which differ among populations and countries [48,49,50]. Such differences in genetic background as well as in environmental triggers could induce different pathways of peripheral aggregation and accumulation of α-synuclein within RBCs. Moreover, previous studies [20,21,22,23,24,25,26] employed homebrew assays, with possible differences in sensitivity and specificity compared to the commercial kits used in our study.

This study has large novelty, since we investigated for the first time the presence of α-synuclein in RBCs in PSP. Moreover, we simultaneously assessed total α-synuclein as well as the oligomeric and phosphorylated pathological variants.

On the other hand, our study displays some limitations. First, as pilot study, we enrolled a limited number of patients, especially in the PSP group, although this low number is not infrequent across the studies in the field due to the rarity of the disease [28,29,42,51,52]. We acknowledge that this could affect the reproducibility of our results and that they must be confirmed in larger cohorts. Moreover, our healthy control cohort was not balanced in terms of female/male ratio. Although we took into account such imbalance by correcting all the comparisons for age and sex, we cannot exclude that it might have a certain impact on our results. Overall, we acknowledge that this is a pilot study and that further work in the field is needed to confirm and extend our findings. Second, patient diagnoses were based only on clinical assessments and pathological confirmation was missing. Although diagnoses were made by movement disorder specialists with more than 10 years of experience and were based on international criteria [1,26], some misdiagnoses might have occurred. Third, due to the low numbers in our groups, we did not further stratify patients based on disease severity and/or subtype.

## 5. Conclusions

In this study, we analyzed RBC total, oligomeric, and p129-α-synuclein levels in PSP for the first time, reporting that RBC total α-synuclein effectively distinguished PSP patients from PD patients, with a good diagnostic accuracy and a positive correlation with disease severity. Although these findings need to be confirmed in a larger cohort, we identified a potential biomarker that could support differential diagnoses and clinical staging of this rare, atypical parkinsonism.

## Figures and Tables

**Table 1:** Demographic and clinical features of PSP and PD patients as well as HCs. Data are shown as mean ± SD.

	PSP (*n* = 8)	PD (*n* = 19)	HC (*n* = 18)	*p*-Value
Sex (F/M)	3/5	7/12	14/4	0.026 ^a^
Age at examination (years)	70.3 ± 6.88	68.8 ± 5.86	66.7 ± 5.12	0.30 ^b^
Disease duration (years)	4.5 ± 2.20	6.5 ± 4.63	-	0.26 ^c^
MDS-UPDRS-III	49.4 ± 17.17	21.3 ± 13.10	-	<0.001 ^c^
PSP Rating Scale	51.0 ± 20.31	-	-	-
HY Staging Scale	3.9 ± 0.83	1.9 ± 0.97	-	<0.001 ^d^

PSP: progressive supranuclear palsy; PD: Parkinson’s disease; HC: healthy control; MDS-UPDRS-III: MDS—Unified Parkinson’s Disease Rating Scale part III; HY: Hoehn and Yahr, ^a^ Fisher’s exact test; ^b^ ANOVA; ^c^ Student’s unpaired *t*-test; ^d^ Mann–Whitney test.

**Table 3:** Correlation analysis of total, oligomeric, and p-129-α-synuclein with demographic and clinical variables in PSP and PD patients and with age in HC.

PSP (*n* = 8)			Age	Disease duration	PSP Rating Scale	MDS- UPDRS-III	HY Staging Scale
	total α-synuclein	Spearman’s rho	0.07	0.15	0.90	-	-
		*p*-value	0.87	0.72	*0.037*	-	-
	oligomeric α-synuclein	Spearman’s rho	0.10	−0.32	0.70	-	-
		*p*-value	0.82	0.44	0.18	-	-
	p129 α-synuclein	Spearman’s rho	−0.67	0.13	0.10	-	-
		*p*-value	0.07	0.76	0.87	-	-
PD (*n* = 19)	total α-synuclein	Spearman’s rho	−0.29	−0.33	-	0.16	−0.21
		*p*-value	0.26	0.22	-	0.56	0.43
	oligomeric α-synuclein	Spearman’s rho	−0.26	−0.12	-	−0.18	−0.31
		*p*-value	0.28	0.63	-	0.48	0.22
	p129 α-synuclein	Spearman’s rho	−0.32	0.15	-	−0.29	−0.11
		*p*-value	0.18	0.54	-	0.24	0.65
HC (*n* = 18)	total α-synuclein	Spearman’s rho	0.19				
		*p*-value	0.44				
	oligomeric α-synuclein	Spearman’s rho	0.10				
		*p*-value	0.69				
	p129 α-synuclein	Spearman’s rho	−0.02				
		*p*-value	0.93				

**Table 2:** Erythrocytic total, oligomeric, and p129-α-synuclein in PSP patients, PD patients, and HCs. Data are corrected for age and sex, and shown as mean ± standard error.

	PSP (*n* = 8)	PD (*n* = 19)	HC (*n* = 18)	*p*-Value
Total α-synuclein (ng/mg)	105.2 ± 5.30 *°	85.5 ± 3.72	88.0 ± 3.74	0.011 ^a^
Oligomeric α-synuclein (ng/mg)	1.10 ± 0.262	1.09 ± 0.171	0.70 ± 0.183	0.29 ^a^
p129-α-synuclein (ng/mg)	1.48 ± 0.216 ^#^	1.22 ± 0.141	0.88 ± 0.151	0.086 ^a^

PSP: progressive supranuclear palsy; PD: Parkinson’s disease; HC: healthy control. ^a^ ANCOVA with age and sex as covariates followed by Tukey’s LSD post hoc test. * PSP vs. PD (*p*-value: 0.004) ° PSP vs. HC (*p*-value: 0.014). ^#^ PSP vs. HC (*p*-value: 0.034). The α-synuclein level (ng) was normalized by Hb level (mg).

## Data Availability

Due to privacy restrictions, the data supporting the results of this study are not publicly available and can be reasonably requested from the corresponding author.

## Supplementary Materials

The following supporting information can be downloaded at: https://www.mdpi.com/article/10.3390/biomedicines12112510/s1, Figure S1: Erythrocytic Hb concentration.
