# Supplementary material for: Erythrocytic α-Synuclein in Parkinson’s Disease and Progressive Supranuclear Palsy—A Pilot Study
Source: Biomedicines. 2024 Nov 2;12(11):2510. doi: 10.3390/biomedicines12112510 (PMC11592387; doi:10.3390/biomedicines12112510)
Supplement: Supplementary file 1 [file biomedicines-12-02510-s001.zip › biomedicines-3278620-supplementary.pdf]

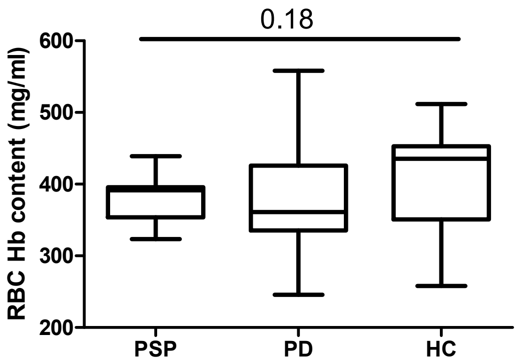

Figure S1. Erythrocytic Hb concentration in PSP (n = 8), PD (n = 19) and HC (n = 18). Data are summarized as box plots, in which the lower, upper, and middle lines of boxes represent the 25th percentile, 75th percentile and median values, respectively, while limits of vertical lines indicate ranges. The p-value was obtained by ANCOVA with age and sex as covariates. o-α-synuclein = oligomeric α-synuclein; PSP = progressive supranuclear palsy; PD = Parkinson's disease; HC = healthy controls.
